# EEG correlates of egocentric and altercentric biases in forensic cases with borderline personality

**DOI:** 10.3389/fnins.2025.1583050

**Published:** 2025-07-10

**Authors:** Vincent Rochas, Marie-Louise Montandon, Cristelle Rodriguez, François R. Herrmann, Ariel Eytan, Alan J. Pegna, Christoph M. Michel, Panteleimon Giannakopoulos

**Affiliations:** ^1^M/EEG & Neuromod Platform, Fondation Campus Biotech Geneva, Geneva, Switzerland; ^2^Department of Rehabilitation and Geriatrics, Geneva University Hospitals, University of Geneva, Geneva, Switzerland; ^3^Division of Institutional Measures, Medical Direction, Geneva University Hospitals, Geneva, Switzerland; ^4^Faculty of Medicine of the University of Geneva, Geneva, Switzerland; ^5^School of Psychology, University of Queensland, Brisbane, QLD, Australia; ^6^Functional Brain Mapping Laboratory, Department of Fundamental Neuroscience, University of Geneva, Geneva, Switzerland

**Keywords:** EEG, perspective taking, altercentric bias, egocentric, borderline

## Abstract

People tend to consider others’ perspective when judging their own (altercentric interference, AI) or other (egocentric interference, EI) divergent views. Borderline (BDL) and antisocial personalities are associated with significant changes in EI and AI. Combining the dot perspective-taking task with high-density EEG recordings, our study explores the correlations between EI and AI in cases with BDL diagnosis and court-ordered measures (BDL-COM; *n* = 14) compared to age-matched healthy controls (*n* = 24). In Inconsistent trials, controls displayed significant activation of brain generators, which was absent in BDL-COM patients. For the Self-Inconsistent stimuli (altercentric bias), controls showed increased activity in the left superior frontal gyrus between 58 and 74 ms and the left inferior frontal gyrus between 279 and 303 ms. Similar differences were observed for Other-Inconsistent stimuli (egocentric bias) in the precentral gyri and inferior frontal gyrus between 274 and 296 ms. These findings suggest that AI involves an early activation of brain generators in central executive and mentalizing areas. EI is associated with an increased activation of the mirror neuron system based on self–other distinction. These EEG data indicate that BDL-COM patients display significant difficulties activating all of the brain generators involved in the processing of conflicting viewpoints in visual perspective-taking.

## Introduction

Neurotypical children and adults over the age of 6 are more likely to experience systematic difficulties caused by a conflict between their own (self) and another’s (other) perspective. Previous studies have shown that people automatically take into account others’ perspectives even when it prevents them from achieving their own goals (Marshall et al., 2018; Qureshi, 2018; Qureshi, 2020). Some observations using the dot perspective-taking developed by Samson et al. (2010) indicated that participants display egocentric bias, referred to as interference, in which their judgment of what an avatar sees is slower or more error-prone when it differs from what participants themselves see Qureshi (2020), Royzman et al. (2003), Wellman (2014), Surtees and Apperly (2012), and Surtees et al. (2016). More recent contributions revealed that egocentric interference (EI) is affected by demographic factors. For instance, male gender and age over 40 are associated with increased egocentric interference (Weidema, 2023; Bradford, 2023). Participants also took longer to report the number of dots they saw in inconsistent trials, where the avatar saw a different number of dots. Altercentric interference (AI) refers to the decreased ability of the participants to report their own perspective when divergent from that of the avatar (Qureshi, 2010; Samson et al., 2010; Marshall et al., 2018; Surtees and Apperly, 2012; Surtees et al., 2016). Altercentric bias is modulated by the age of the avatar rather than sex (Ferguson et al., 2018). Moreover, adults over the age of 55 years show increased sensitivity to others’ conflicting viewpoints, as documented by increased reaction time in case of incongruence when adopting their own perspective (Martin, 2019). Importantly, a recent study suggested that persons with lower attentional resources and higher impulsivity are less responsive to both egocentric and altercentric biases (Rodriguez et al., 2022).

Although there is an ongoing theoretical debate about whether automatic interference effects in the dot perspective task reflect the activation of domain-specific perspective-taking processes or domain-general submentalizing processes [for review, see Marshall et al. (2018), Ferguson et al. (2018), Cole and Millett (2019), and Westra et al. (2021)], recent evidence suggests that this human ability may be of key importance for social interactions as well as in clinical settings (Furlanetto et al., 2016; Drayton et al., 2018; Gao et al., 2019; Del Sette et al., 2022). In an early study, Drayton et al. (2018) reported that offenders from a high-security hospital with high levels of psychopathy can represent others’ perspectives in goal-conducive tasks but show a striking ability to ignore them in non-goal-relevant situations. Later, similar reports were reported in patients with autism spectrum disorders (Doi et al., 2020). This resistance to AI could partly explain the maladaptive social behavior of these patients. In contrast, patients with borderline personality disorders (BPDs) are considered to be more sensitive to both egocentric and altercentric biases due to their struggles with differentiating self from others (De Meulemeester et al., 2021).

The neural and functional bases of EI and AI is still a matter of debate. Previous studies indicated that the dorsolateral prefrontal cortex (PFC), as well as the more posterior and dorsal parts of the frontal cortex and temporo-parietal junction, may be involved in consistency contrast to others’ perspectives [egocentric bias; Bukowski, 2018]. Using the dot perspective-taking task, we recently postulated that the fMRI correlates of EI should include parts of the mirror neuron system (middle and superior precentral gyri) and the left frontopolar cortex (Montandon et al., 2023). In an early study, Schurz (2018) found that AI activated the right temporo-parietal junction and ventral medial prefrontal cortex (PFC). In a recent study, we found that AI was associated with increased activation in the lateral occipital cortex, right supramarginal and angular gyrus, and the inferior, superior, and middle frontal gyri (Montandon et al., 2023). These studies focused only on healthy controls and, due to the inherent limitations of MRI scans, did not address the temporal processing of brain generators involved in these implicit biases. To date, no study has examined the electrophysiological correlates of EI and AI in clinical samples. Coupling the dot perspective-taking task with high-density EEG recordings at the surface and in the inverse space to define the brain sources of electrical activity, the present study investigates the differences in the EEG activation of neural generators in the context of EI and AI between forensic cases with BDL diagnoses and court-ordered measures for criminal offenses (BDL-COM) and age-matched healthy controls without a history of previous convictions.

## Materials and methods

### Population

The study protocol was approved by the local Ethics Committee [Commission cantonale d’éthique de la recherche (CCER), decision 2019–00794], and all participants provided written informed consent before initial inclusion. All control cases were recruited through advertisements in local newspapers and media. A total of 15 patients were recruited among those who were regularly followed-up for COM in the Service of Institutional Measures, a specialized division in charge of COM at the University Hospitals of Geneva. All methods were conducted in accordance with the World Medical Association Declaration of Helsinki and the Principles of Good Clinical Practice. All participants underwent a detailed psychiatric assessment conducted by a board-certified fully trained psychiatrist (AE). They were evaluated using the PCL-R (Psychopathy Checklist Revised), which is a pivotal tool for identifying psychopathic individuals in correctional settings (Coid et al., 2009). The exclusion of acute psychiatric disorders was confirmed by the Mini Neuropsychiatric Interview (Doyle and Dolan, 2006). The BDL diagnosis was extracted from psychiatric expert assessments using ICD-10 criteria (World Health Organization, ICD-10: International statistical classification of diseases and related health problems: Tenth revision, second ed. Geneva: World Health Organization). Subsequently, it was confirmed by the assessment made by the board-certified psychiatrist. In case of disagreement, candidate cases were excluded from further studies. They were also excluded if they had a history of loss of consciousness lasting longer than 15 min, a head injury or post-concussion symptoms, seizure and neurological disorders, regular use of psychotropic medication, and uncorrected auditory or visual deficits. Finally, 15 patients and 24 healthy participants were included in the EEG part of this study.

### Dot perspective-taking task

The dot perspective-taking (dPT) task used in this study was initially adapted from Samson et al. (2010) and modified from a previous EEG study (Rochas et al., 2023). The task consisted of the presentation on an LED screen of a picture of a scene of an avatar in the middle of a square room and looking in one direction, either left or right. The shown room has one to three red dots distributed on two side walls. The trials can be consistent or inconsistent in terms of the number of dots seen by the participant in the entire picture and by the avatar in the scene on the wall in front of him. During the scene display, the participant had to respond if the cued number corresponded to the number of dots actually seen from the cued perspective, i.e., himself or the avatar. The task was presented using E-Prime 3.0 software (Psychology Software Tools, Pittsburgh, PA). One trial consisted of a fixation cross for 750 ms, a perspective cue for 1,000 ms, and a number cue for 1,000 ms that indicates the number of dots to be seen from 0 to 3. After that, another fixation cross was displayed for a random duration between 400 and 500 ms, followed by a picture of the scene for 2000 ms, during which the correct or incorrect response was taken by pressing a button on a response box with the dominant hand. The task is delivered in 3 blocks of 144 trials, allowing for some rest during breaks. In total, there were 96 consistent trials with the Self-perspective, 96 consistent trials with the Other-perspective, 96 inconsistent trials with the Self-perspective, and 96 inconsistent trials with the Other-perspective. Additionally, 48 trials with no dots at all and, by definition, consistency were also displayed, 24 with the Self-perspective and 24 with the Other-perspective. For each condition, half of the trials were correct, while the other half were incorrect.

### EEG acquisition

The EEG signal was recorded using an EGI GES 400 amplifier at a sampling rate of 1,000 Hz and a high-density EGI 256 electrode Hydrocel Geodesic Sensor Net referenced to the Cz electrode. The impedances of the electrodes were kept below 30 kOhms during the sessions. The acquisition took place in a dark and soundproofed Faraday cage, with participants positioned on a chinrest situated 80 cm from the screen in order to perform the task in quiet conditions.

### EEG preprocessing

The EEG signal preprocessing was performed using Cartool 4.13 (Brunet et al., 2011; Michel and Brunet, 2019) (https://sites.google.com/site/cartoolcommunity/; https://github.com/DenisBrunet/Cartool). First, the originally recorded 257 channels were restricted to 204, excluding the channels corresponding to the cheeks and neck electrodes. The signal was filtered with a DC removal, a bandpass Butterworth filter from 1 to 80 Hz, a Butterworth notch filter at 50 Hz, and all possible harmonics. Before further analysis, the recordings were then reviewed (by VR) for removal of periods with large movement artefacts and bad channels (199 channels kept on average). Using runICA from EEGlab (Delorme and Makeig, 2004) in Matlab, an independent component analysis was performed on the data to identify components related to non-EEG signals (eye saccades and blinks, cardiac interference, and neck or jaw muscle tension). These bad components were discarded from the reconstructed data for further analysis. The signals from the channels identified as bad were interpolated using a 3D spline method in Cartool software.

### Event-related analyses

Time epochs were isolated from −500 to +1,500 ms relative to the scene picture onset separately for the four different conditions: Consistent with Self-perspective, Consistent with Other-perspective, Inconsistent with Self-perspective and Inconsistent with Other-perspective. The number of epochs was equally adjusted between conditions individually (i.e., random picking of the number for the lowest condition for each participant). On average, 88.9 epochs (standard deviation = 6.4) were considered for further analysis. The clean EEG epochs were averaged per participant and per condition to compute surface event-related potentials (ERPs). To characterize the sources of the differences observed on the surface, event-related source reconstruction was also computed in Cartool (Michel and Brunet, 2019). First, the epoch data underwent spatial filtering considering the position of the electrodes on the scalp surface. The inverse model used an MNI template head, 6,008 solution points symmetrically distributed in the grey matter, and an EGI net model corresponding to the 204 selected electrodes co-registered on the template head. A lead field was calculated for the four shells (scalp, skull, CSF, and brain) of the segmented template using the Locally Spherical Model with Anatomical Constraints (LSMACs). This exact spherical equation was used to calculate a distributed linear inverse solution, LORETA, between the 204 electrodes and the 6,008 solution points. Finally, individual normalization using the background activity from the results of the inverse solution of the whole epoch data was used to estimate a baseline and a scaling factor for each solution point. We obtained individual normalized event-related source reconstructions in scalar values for the four different conditions.

### Statistics

Fisher’s exact tests were performed to compare sociodemographic (age and years of education) and clinical variables (PCL-R score) between controls and BDL-COM patients. To investigate behavioral aspects, mixed-design ANOVAs were computed on accuracies (i.e., percentage of correct responses) and reaction times with the within-subject factors consistency (Consistent vs. Inconsistent) and perspective (Self vs. Other), and the between-subject factor group (Patients vs. Controls). Surface ERPs were analyzed using the randomization statistic toolbox RAGU (Koenig et al., 2011; Habermann et al., 2018), for details on statistical principles. A topographic consistency test, based on the comparison of the grand-mean global field power (GFP) of original data against the grand-mean GFP of shuffled maps, was conducted to define periods of consistent neural activation across subjects for a given condition. Hence, further analyses were restricted to the period of time of consistency across all conditions. ANOVAs were used on the global field power (GFP) to compare differences among the three factors for original data and condition-randomized data, with consistency and perspective as within-subject factors and the patient-control group as a between-subject factor. Similarly, a three-by-two ANOVA with the same factor design was conducted on topographies using the GFP of the delta map to reveal differences in the distribution of neural processing. To assess the validity of significance, the distribution of all obtained *p*-values for all randomization runs was combined (Global p-AUC Statistics) using Fisher’s method (Fisher, 1925) and compared to the original data p-values. To understand the substrate of the surface differences, event-related sources were reconstructed for the four different conditions and two groups, and the results were tested between groups using unpaired t-test statistics with false discovery rate (FDR) correction.

## Results

### Behavioral performance

After the exclusion of an outlier (performance of zero for the Self Inconsistent trials, 90% accuracy in the other conditions), the final sample included 24 control participants (mean age: 42.05 ± 3.51 years, education: 14.60 ± 5.70 years) and 14 BDL-COM patients (mean age: 40.01 ± 4.92 years, education: 13.90 ± 6.70 years). There were no significant group differences in age and years of education. Although clearly below the cutoff for psychopathy (total score > 24), the PCL-R score of the BDL-COM group was significantly higher than that of controls [7.87 (5.26) vs. 2.39 (3.65); *p* < 0.001].

**Figure 1:**
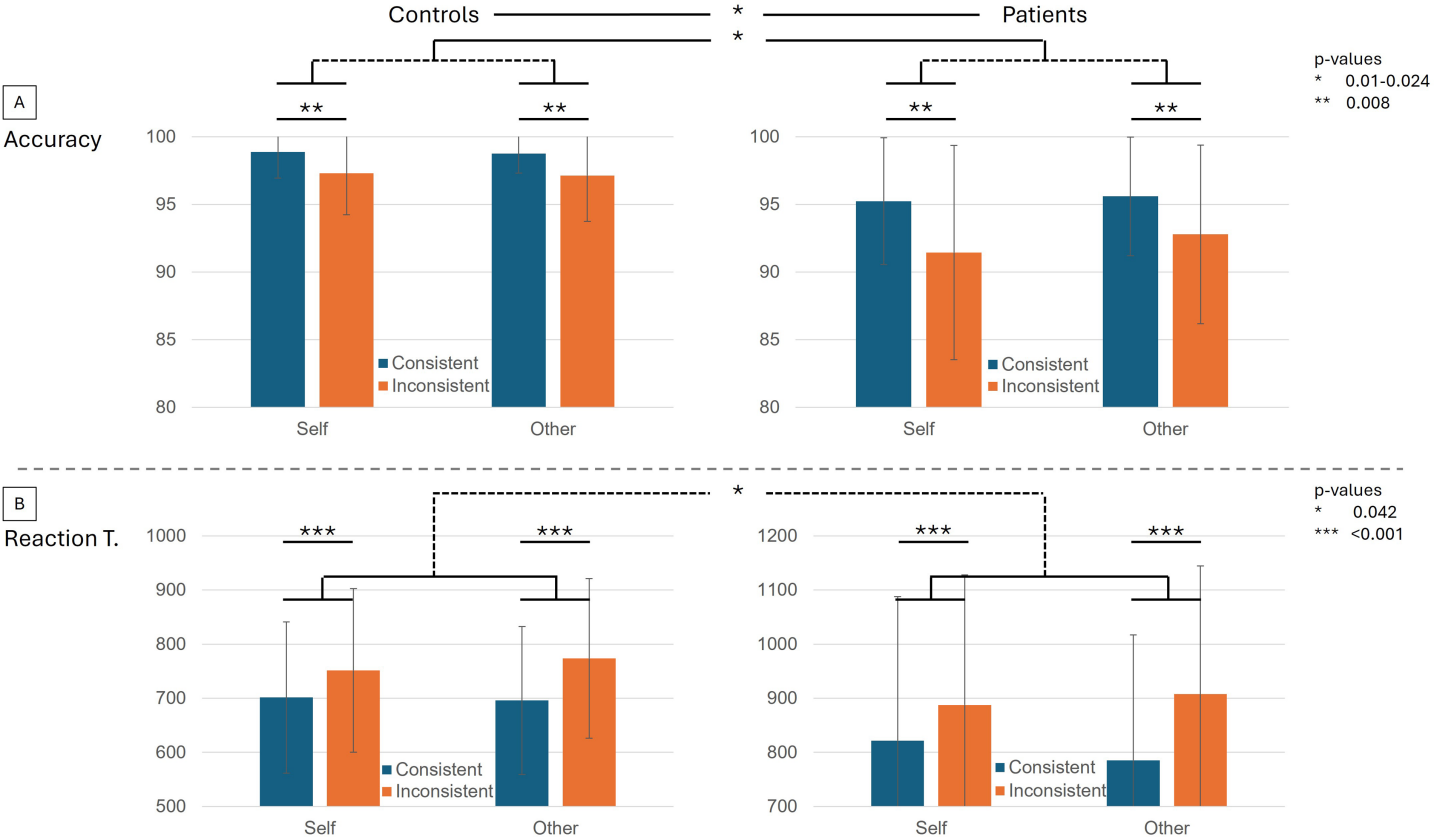
Behavioral performances in the dot perspective task. **(A)** The upper bar plots display the average accuracy in controls and BDL-COM patients. Within the plots, the performances are distributed between the four conditions of the task, self and other-perspective from left to right, Consistent in blue and Inconsistent in orange. **(B)** Average reaction times in controls and BDL-COM patients. Within plots, the performances are distributed between the 4 conditions of the task, self and other-perspective from left to right, Consistent in blue and Inconsistent in orange. Error bars represent standard deviations. Significant differences and interactions are indicated with asterisks corresponding to *p*-value ranges.

The performance in the task was very high, with more than 90% accuracy over all conditions and groups (Table 1). Inconsistent trials were less well processed according to the mixed design ANOVA with decreased accuracy (*z* = −2.651, *p* = 0.008) and increased reaction time (*z* = 5.222, *p* < 0.001) than Consistent trials (Figure 1). A significant perspective x consistency interaction (*z* = 2.035, *p* = 0.042) showed that reaction times were significantly higher in Inconsistent trials with the Other-perspective. Overall, BDL-COM patients showed decreased accuracy (*z* = −2.589, *p* = 0.01) and a tendency to increased reaction times (*z* = 1.920, *p* = 0.055) compared to control participants. There was a more marked decrease of accuracy in Self-perspective trials, with a significant interaction between Perspective x Group interaction (*z* = −2.256, *p* = 0.024). Similarly, BDL-COM patients showed significantly longer reaction times in Other-perspective trials than controls, with a significant Perspective x Group interaction (*z* = −1.957, *p* = 0.050).

**Table 1:** Dot perspective task performances, with accuracy in % of correct answers (left), and reaction times in ms (right) for control participants and BDL-COM patients.

		Accuracy				Reaction times			
		Consistent self	Inconsistent self	Consistent other	Inconsistent other	Consistent self	Inconsistent self	Consistent other	Inconsistent other
Control participants	Average	98.9	97.3	98.8	97.1	701	751	696	774
	Std Dev	1.9	3.1	1.4	3.4	140	151	137	148
BDL-COM	Average	95.2	91.4	95.6	92.8	822	888	785	907
	Std Dev	4.7	7.9	4.4	6.6	266	240	232	237
Total	Average	97.5	95.1	97.6	95.5	746	802	729	823
	Std Dev	3.6	6.0	3.2	5.2	201	197	180	194

Performances are given in average with its standard deviation.

### Event-related analyses

#### Scalp level

**Figure 2:**
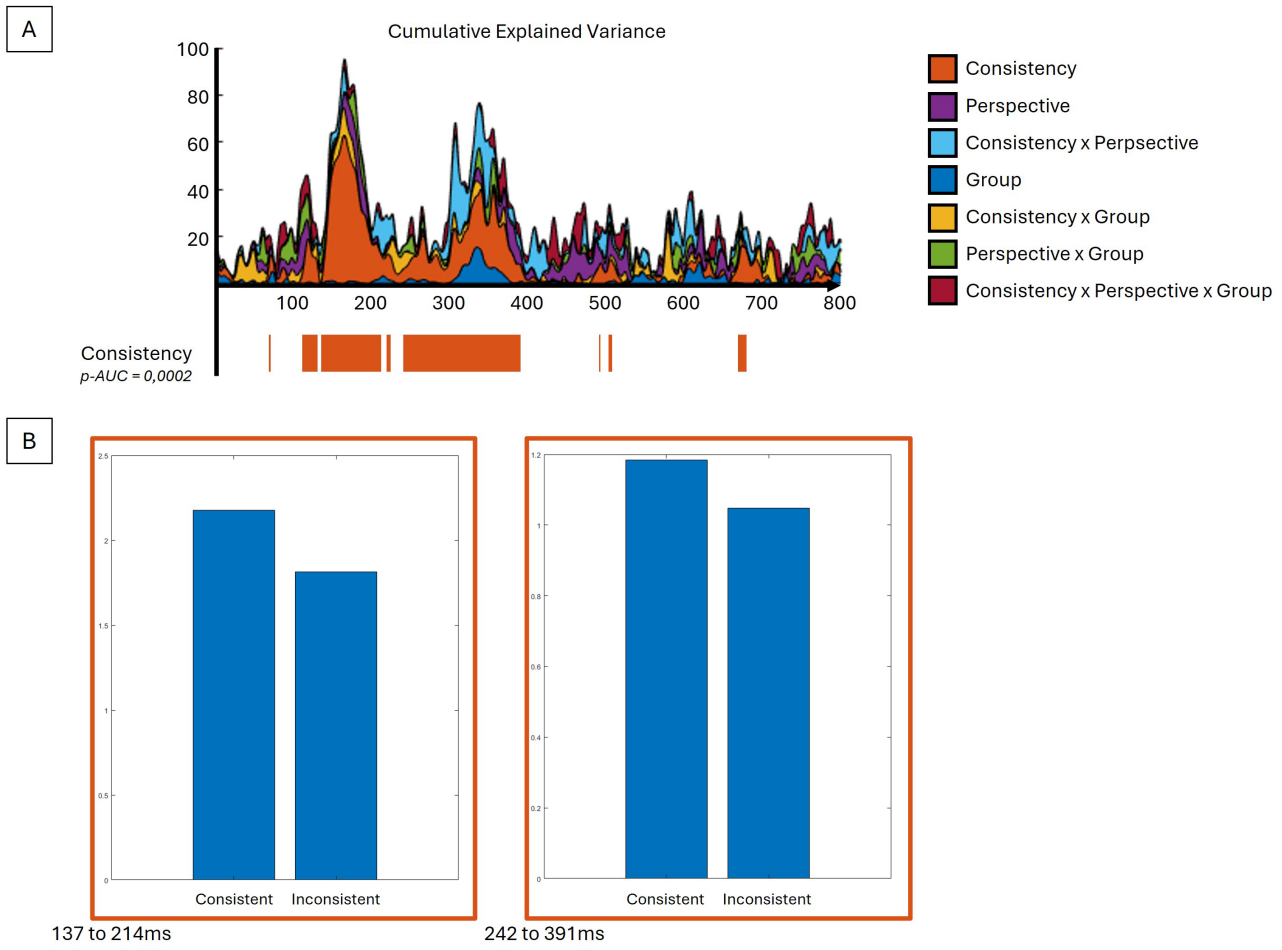
GFP analyses reveal differences in EEG intensity. **(A)** The upper graph displays the explained variance percentage curves according to the different contrasts and their sum in cumulative explained variance. In its lower part, the significant time periods for the consistency contrast are depicted along the same time axis. **(B)** The bar plots show the direction difference between the conditions of the consistency contrast averaged for significant time periods. See text for details.

**Figure 3:**
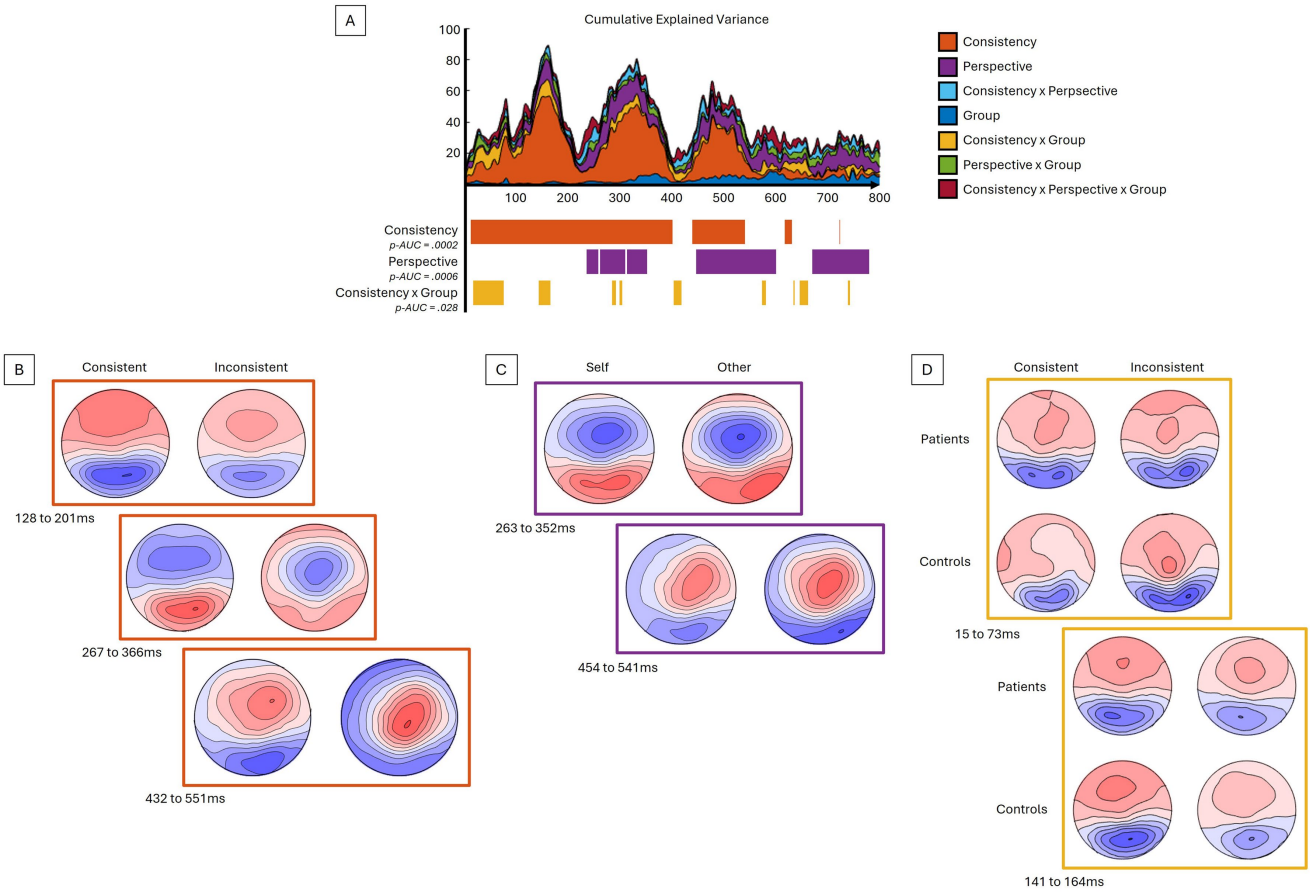
Topographical ANOVA reveals differences in EEG configuration. **(A)** The upper graph displays the explained variance percentage curves according to the different contrasts and their sum in cumulative explained variance. In its lower part, the significant time periods for the given contrasts are depicted along the same time axis. **(B–D)** The topographical maps display the difference in the distribution of voltage potential between conditions; **(B)** for consistency, **(C)** for perspective, and **(D)** for interaction between consistency and group – averaged for different time periods. See text for details.

Taking into account the four conditions and two groups, the period of relative topographic consistency between stimulus onset and 800 ms post-stimulus was selected. This period was used to analyze the event-related response analyses at the scalp level and in the inverse space. When compared to Inconsistent trials, the Consistency factor showed significant differences (*p-AUC* = 0.0002), with a stronger global field power (GFP) response, particularly for the Consistent trials, notably over the N170 (137-214 ms) and P300 (242 to 391 ms) components (Figure 2). With respect to topographies (Figure 3), the Consistency factor showed significant differences during the first 550 ms, with topographies more pronounced on the anteroposterior axis for Consistent compared to Inconsistent trials. This was the case for the three main visual components, P100 (not depicted, max EV = 27.16%), N170 (max EV = 55.10%), and P300 (max EV = 46.14%), as well as the late 500 ms component (max EV = 38.81%). Later differences in topography were observed with respect to the Perspective factor, with less centred and rightward topographies for the Other- compared to Self-perspective during the P300 (max EV = 16.19%), 500 ms (max EV = 13.30%), and also as late as 700 to 800 ms (max EV = 14.90%) components. Significant interactions between the factors Group and Consistency were presented very early (15 to 73 ms) (max EV = 9.78% at 65 ms) and later on (141 to 164 ms). BDL-COM patients did not exhibit the topographical differences of neural generators as a function of the Consistency factor that were observed in controls.

#### Inverse solutions

**Figure 4:**
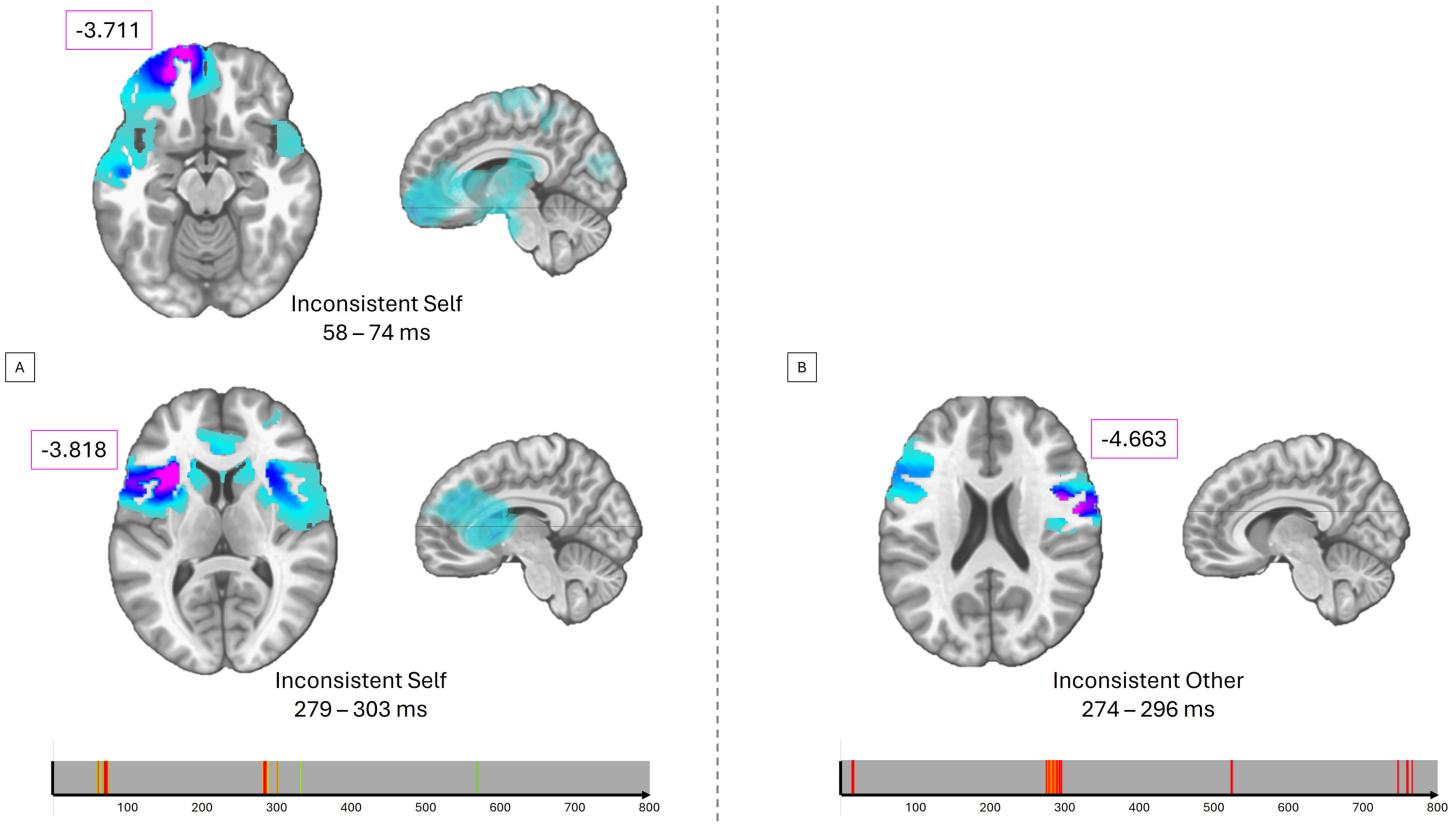
Differencing source estimations. **(A)** *T*-values for significant group differences for the inconsistent self-perspective condition averaged over different time periods. The lower timing plots represent the significant *p*-values as 1-p from green to red. **(B)** *T*-values for significant group differences for the inconsistent other-perspective condition averaged on a given time period.

For the Inconsistent stimuli with Self-perspective (altercentric bias), control participants showed increased activity compared to BDL-COM patients in the left superior frontal gyrus (BA11) between 58 and 74 ms and in the left inferior frontal gyrus and insula (BA13) between 279 and 303 ms (Figure 4A). The same pattern of group differences was observed for Inconsistent stimuli with Other-perspective (egocentric bias) in the right insula, precentral gyrus, and inferior frontal gyrus (BA13, 44, 6) between 274 and 296 ms (Figure 4B).

## Discussion

Our analysis focuses on BDL cases with court-ordered probation or outpatient treatment after criminal convictions. Although rare in psychiatric practice, these patients are of particular interest in studies of human empathy because they are known to suffer from increased impulsiveness as well as impaired mentalization abilities (Lenzen et al., 2021; Nasello et al., 2023; Normann-Eide et al., 2020). Both the resistance to altercentric bias reported in cases with high levels of psychopathy (Drayton et al., 2018) and the increased vulnerability to both egocentric and altercentric biases in BDL patients (De Meulemeester et al., 2021) highlight further points the interest of this clinical sample when exploring the neural substrates of perspective-taking.

### Behavioral data

In terms of mean reaction times, inconsistency was associated with a significant increase in the occurrence of errors both in Self and Other conditions, confirming the presence of both altercentring and egocentring biases in our sample (Samson et al., 2010; Qureshi, 2010; Montandon et al., 2023). Overall, BDL-COM patients showed decreased accuracy in Self-perspective trials and increased reaction times in Other-perspective trials compared to controls. However, the absence of a significant Group X Consistency interaction does not support the idea of an increased sensitivity of BDL-COM patients to both types of interference. Of note, the present sample of patients includes a BDL subtype with significantly increased PCL-R scores than the controls. One could speculate that this relative increase of the levels of psychopathy provides protection, limiting both the AI and EI in perspective-taking (Drayton et al., 2018; Song, 2023).

### Quantitative EEG findings

Using high-density EEG, we report the patterns of activation of brain generators related to egocentric and altercentric biases in BDL-COM patients and healthy controls. To the best of our knowledge, this is the first study exploring the EEG correlates of these biases in a forensic sample. As reported previously (Rochas et al., 2023), and in line with the longer reaction times observed for other-inconsistent trials, GFP values were significantly higher for the Other-compared to the Sel-perspective after 300 ms post-stimulus and even in very late points (700 to 800 ms). This reflected the need for additional brain effort when adopting the other perspective independently of the clinical diagnosis.

Our observations are also consistent with the fMRI observations by Montandon et al. (2023) who reported that the Other is a much more expensive brain process compared to the self perspective and relies on the activation of brain areas involved in theory of mind, such as the precuneus and superior parietal cortex, as well as the salience network and decision-making areas. Consistent trials were associated with stronger GFP responses than Inconsistent stimuli, which concerned mainly the visual components P100, N170, and P300. Early visual processing for the detection of similarities has already been reported in face encoding (Heisz, 2006; Böckler and Zwickel, 2013), and familiarity in the perspective calculation process (Saether et al., 2021) may partly explain this difference. The Group x Consistency interaction was significant at early time points (15 to 73 ms, 141 to 164 ms), with a marked difficulty for BDL-COM patients in the mobilization of additional neural resources according to the consistency of the stimulus.

### Inverse space analysis

The inverse space analysis revealed marked differences in the activation of the neural generators involved in EI and AI between BDL-COM cases and controls. In Self Inconsistent trials, contrast to AI, controls displayed an early increase in the activation of brain generators in the left superior frontal gyrus (58 to 74 ms), followed by the left inferior frontal gyrus and insula (279 to 303 ms). In Other inconsistent trials, which correspond to EI, this increased activation occurred between 274 and 296 ms, mainly in the right insula, precentral gyrus, and inferior frontal gyrus. These EEG observations complete the fMRI data reported by Montandon et al. (2023) by including temporal processing of brain activation related to both types of interference. In particular, they indicate that AI is related to an early activation of brain generators in central executive areas such as the superior frontal gyrus and inferior frontal gyrus, which are thought to be engaged whenever a task-relevant perspective needs to be selected over an irrelevant one (Ramsey et al., 2013). In terms of EI, the activation of neural generators in precentral areas indicates the involvement of the mirror neuron system for action understanding based on self-other distinction, which is coupled with that of the inferior frontal gyrus in assessing the validity of the others’ viewpoint (Coetzee and Monti, 2018). BDL-COM patients displayed significant difficulties in the activation of all of these brain generators in line with their well-known difficulty of this population when addressing the self-other distinction (De Meulemeester et al., 2021). It should be noted, however, that despite these EEG deficits, our BDL-COM patients did not show an increased vulnerability to Inconsistency when compared to controls, as documented by our behavioral data, indicating the presence of brain compensatory mechanisms that protect the cognitive performance.

### Strengths and limitations

Strengths of the present study include the use of a well-documented clinical sample and the combination of topographic analysis and inverse space solutions. However, there are several limitations to consider. The male sample, the exclusion of neurological and acute psychiatric disorders, regular use of psychotropics and active substance abuse limit the generalizability of our observations. Second, in the absence of previous observations in this field, we cannot exclude that the case–control differences reported here partly reflect the selection of forensic BDL cases and are not representative of the whole spectrum of this pathology. Future studies in larger samples of forensic and non-forensic BDL patients, including women, with a larger diagnostic spectrum (antisocial personality, attention deficits), and combining several imaging modalities (fMRI, single photon emission computerized tomography) are warranted to get better insight into the visual perspective taking-related deficits in brain activation in forensic samples.

## Conclusion

In conclusion, this first analysis of the EEG correlates of EI and AI in a forensic sample of BDL patients identified decreased activation of neural generators in both the central executive and mentalizing areas responsible for AI and the mirror neuron system for EI, as compared to controls. These early EEG changes are not associated with decreased cognitive performance in visual perspective taking. They could be used in larger clinical samples to explore the presence of early functional alterations related to EI and AI in other groups of forensic patients.

## Data Availability

The raw data supporting the conclusions of this article will be made available by the authors, without undue reservation.
